# Effects of glycyrrhizin on the pharmacokinetics of asiatic acid in rats and its potential mechanism

**DOI:** 10.1080/13880209.2018.1428634

**Published:** 2018-01-22

**Authors:** Ling Guo, Ying Cui, Kaijun Hao

**Affiliations:** a Department of Nursing, Yidu Central Hospital of Weifang, Shandong, China;; b Qingzhou Hospital for Disabled Soldiers, Shandong, China

**Keywords:** Drug–drug interaction, P-gp, CYP450

## Abstract

**Context:** Asiatic acid has been reported to possess a wide range of pharmacological activities.

**Objective:** This study investigates the effects of glycyrrhizin on the pharmacokinetics of asiatic acid in rats and its potential mechanism.

**Materials and methods:** The pharmacokinetics of orally administered asiatic acid (20 mg/kg) with or without glycyrrhizin pretreatment (100 mg/kg/day for seven days) were investigated using a LC–MS method. Additionally, the Caco-2 cell transwell model and rat liver microsome incubation systems were used to investigate the potential mechanism of glycyrrhizin’s effects on the pharmacokinetics of asiatic acid.

**Results:** The results showed that the *C*_max_ (221.33 ± 21.06 vs. 324.67 ± 28.64 ng/mL), AUC_0–inf_ (496.12 ± 109.31 vs. 749.15 ± 163.95 μg·h/L) and the *t*_1/2_ (1.21 ± 0.27 vs. 2.04 ± 0.32 h) of asiatic acid decreased significantly (*p* < 0.05) with the pretreatment of glycyrrhizin. The oral clearance of asiatic acid increased significantly from 27.59 ± 5.34 to 41.57 ± 9.19 L/h/kg (*p* < 0.05). The Caco-2 cell transwell experiments indicated that glycyrrhizin could increase the efflux ratio of asiatic acid from 1.63 to 2.74, and the rat liver microsome incubation experiments showed that glycyrrhizin could increase the intrinsic clearance rate of asiatic acid from 138.32 ± 11.20 to 221.76 ± 16.85 μL/min/mg protein.

**Discussion and conclusions:** In conclusion, these results indicated that glycyrrhizin could decrease the system exposure of asiatic acid, possibly by inducing the activity of P-gp or CYP450 enzyme.

## Introduction

Asiatic acid is a pentacyclic triterpene isolated from *Centella asiatica* (Bian et al. 2013; Bunbupha et al. 2014; Kwon et al. 2014). Asiatic acid is widely used in China for wound healing, various skin conditions, inflammatory, diabetic, hyperlipidaemic, and also for antidepressant, relieving anxiety, and improving cognition (Kavitha et al. 2011; Lee et al. 2012; Bylka et al. 2014; Luo et al. 2014; Pakdeechote et al. 2014; Li et al. 2016). Previous studies have reported that the oral bioavailability of asiatic acid is very low, and therapeutic efficacy of asiatic acid is restricted due to its low penetrability into the brain or other target organ as well as the efflux mediated by P-gp (Chassaud et al. 1971; Abuznait et al. 2011; Nair et al. 2012; Yuan et al. 2015).

Liquorice is the root of *Glycyrrhiza uralensis* Fisch. or *Glycyrrhiza glabra* L. (Leguminosae). Liquorice has been commonly used together with other herbs to enhance the effects of other ingredients or to reduce toxicity in traditional Chinese medicine (Wang et al. 2013; Link et al. 2015). Glycyrrhizin, a triterpenoid saponin isolated from licorice, has anti-inflammatory, hepato-protective and antitumor properties (Chen et al. 2017; Jia et al. 2017; Mu et al. 2017). Several research articles have indicated that glycyrrhizin could modulate the activity of CYP3A4 and P-gp, which might lead to drug–drug interactions when they are co-administered with other herbs or drugs (Chen et al. 2009; Yan et al. 2017; Zhao et al. 2017). Chinese medicines are often co-administered in clinical practice with or without patients’ knowledge, which may greatly raise the potential of drug–drug interactions. These interactions can cause significant safety concerns because the pharmacokinetics of the drug and/or the active constituent of Chinese medicines may be altered by co-administration; severe and perhaps even life-threatening adverse reactions may occur (Wang et al. 2015; Li et al. 2016). However, the drug–drug interaction between asiatic acid and glycyrrhizin is still unknown, especially the effects of glycyrrhizin on the pharmacokinetics of asiatic acid.

This study investigates the effects of glycyrrhizin on the pharmacokinetics of asiatic acid in rats and its potential mechanism. The *in vivo* pharmacokinetics of asiatic acid in rats with or without pretreatment with glycyrrhizin were determined using a sensitive and reliable LC–MS method. Additionally, the Caco-2 cell transwell model and the rat liver microsome incubation systems were also used to determine the mechanism of the effect.

## Materials and methods

### Chemicals and reagents

Asiatic acid (purity >98%) and celastrol (purity >98%) were purchased from the National Institute for the Control of Pharmaceutical and Biological Products (Beijing, China). Glycyrrhizin (purity >98%) was obtained from Shanghai Standard Biotechnology Co., Ltd. (Shanghai, China). β-Nicotinamide adenine dinucleotide phosphate (NADP^+^) and lucifer yellow were provided by Sigma (St. Louis, MO). Rat liver microsomes were purchased from BD (Woburn, MA). Dulbecco’s modified Eagle’s medium (DMEM) and non-essential amino acid (NEAA) solution were purchased from Thermo Scientific Corp. (Logan, UT). Foetal bovine serum was obtained from GIBCO BRL (Grand Island, NY). Hanks’ balanced salt solution (HBSS) was purchased from GIBCO (Grand Island, NY). Acetonitrile and methanol were purchased from Fisher Scientific (Fair Lawn, NJ). Ultrapure water was prepared with a Milli-Q water purification system (Millipore, Billerica, MA). All other chemicals were of analytical grade or better.

### Animal experiments

Male Sprague-Dawley rats weighing 230–250 g were provided by the experimental animal center of the Weifang Medical University (Weifang, China). Rats were bred in a breeding room at 25 °C with 60 ± 5% humidity and a 12 h dark–light cycle. Tap water and normal chow were given *ad libitum*. All of the experimental animals were housed under the above conditions for a three-day acclimation period and fasted overnight before the experiments. All experimental procedures and protocols were reviewed and approved by the Animal Care and Use Committee of Weifang Medical University and were in accordance with the National Institutes of Health guidelines regarding the principles of animal care.

### *In vivo* pharmacokinetic study

To evaluate the effects of glycyrrhizin on the pharmacokinetics of asiatic acid, the rats were divided into two groups of six animals each. The test group was pretreated with glycyrrhizin at a dose of 100 mg/kg/day for seven days before the administration of asiatic acid. Next, asiatic acid was orally administered to rats by gavage at a dose of 20 mg/kg. Blood samples (250 μL) were collected into heparinized tubes via the *oculi chorioideae* vein at 0.083, 0.167, 0.33, 0.5, 1, 2, 4, 6 and 8 h after the oral administration of asiatic acid. The blood samples were centrifuged at 3500 rpm for 5 min. The plasma samples that were obtained were stored at –40 °C until analysis.

### Instruments and conditions

The analysis was performed on an Agilent Series 1100 HPLC system and Agilent G1946 single quadrupole mass spectrometer (Palo Alto, CA) as previously reported (Yuan et al. 2015). The chromatographic analysis of asiatic acid and celastrol was performed on a Waters X-Bridge C18 column (3.0 × 100 mm, 3.5 μm) at room temperature. The mobile phase was water (5 mM aqueous ammonium acetate) and acetonitrile (50:50, v:v) at a flow rate of 0.8 mL/min. The mass spectrometric analysis was performed in negative ion mode with a selected ion monitoring (SIM) method. The other parameters were as follows: fragmentor: 170 v; drying gas flowing rate: 11.0 L/min; nebulizer pressure: 40 psi; drying gas temperature: 350 °C; capillary voltage: 3500 V. The selected ion was *m*/*z* 487.3 for asiatic acid and *m*/*z* 449.1 for celastrol.

### Cell culture

The Caco-2 cell line was obtained from the American Type Culture Collection (Manassas, VA). The Caco-2 cells were cultured in DMEM high glucose medium containing 15% FBS, 1% NEAA and 100 U/mL penicillin and streptomycin. The cells were cultured at 37 °C with 5% CO_2_. For transport studies, the cells at passage 40 were seeded on transwell polycarbonate insert filters (1.12 cm^2^ surface, 0.4 μm pore size, 12 mm diameter; Corning Costar Corporation, Cambridge, MA) in 12-well plates at a density of 1 × 10^5^ cells/cm^2^. Cells were allowed to grow for 21 days. For the first seven days, the medium was replaced every two days and daily thereafter. The transepithelial electrical resistance (TEER) of the monolayer cells was measured using Millicell ERS-2 (Millipore Corporation, Billerica, MA). TEER value exceeding 400 Ω·cm^2^ was used for the flux experiment. The integrity of the Caco-2 monolayers was confirmed by the paracellular flux of Lucifer yellow, which was less than 1% per hour. The alkaline phosphatase activity was validated using an Alkaline Phosphatase Assay Kit. The qualified monolayers were used for transport studies.

### Effects of glycyrrhizin on the absorption of asiatic acid in Caco-2 cell transwell model

Before the transport experiments, the cell monolayers were rinsed twice using warm (37 °C) HBSS, and the Caco-2 cell transwell model was incubated at 37 °C for 20 min. After incubation, the Caco-2 cell transwell model was incubated with asiatic acid in fresh incubation medium added on either the apical or basolateral (BL) side for the indicated times at 37 °C. The volume of incubation medium on the apical and BL sides was 0.5 mL and 1.5 mL, respectively. A 100 μL aliquot of the incubation solution was withdrawn at the indicated time points from the receiver compartment and replaced with the same volume of fresh pre-warmed HBSS buffer. The efflux activity of P-gp was validated using a typical P-gp substrate digoxin (25 μM). The effects of glycyrrhizin or verapamil (P-gp inhibitor) on the transport of asiatic acid were investigated by adding 50 μM glycyrrhizin or/and verapamil to both sides of the cell monolayers and preincubating the sample at 37 °C for 24 h. In addition, the effects of glycyrrhizin on the efflux of digoxin (25 μM) were also investigated. The permeability of asiatic acid (5 μM) (which was validated for no toxicity for Caco-2 cells within two hours) in all of the above conditions for both directions, i.e., from the apical (AP) side to the BL side and from the BL side to the AP side, was measured after incubation for 30, 60, 90 and 120 min at 37 °C.

The apparent permeability coefficient (*P_app_*) was calculated using the equation of Artursson and Karlsson:

$$P_{app}=\;\left(\Delta Q/\Delta t\right)\;\times \;\left[1/\left(A\;\times \;C_{0}\right)\right]$$

where *P_app_* is the apparent permeability coefficient (cm/s), Δ*Q*/Δ*t* (μmol/s) is the rate at which the compound appears in the receiver chamber, *C*_0_ (μmol/L) is the initial concentration of the compound in the donor chamber and *A* (cm^2^) represents the surface area of the cell monolayer. Data were collected from three separate experiments, and each was performed in triplicate.

### Effects of glycyrrhizin on the metabolic stability of asiatic acid in rat liver microsome incubation systems

The effects of glycyrrhizin on the metabolic stability of asiatic acid were investigated using rat liver microsome incubation experiments. The assay conditions and reaction mixtures were similar to those reported previously (Wang et al. 2017). In brief, except for NADPH-generating system, 10 μL rat liver microsomes (20 mg/mL), 4 μL asiatic acid solution (100 μM) and 366 μL PBS buffer were added to the centrifuge tubes on ice. The reaction mixture was incubated at 37 °C for 5 min and then NADPH-generating system (15 μL) was added. The effects of glycyrrhizin or verapamil on the metabolic stability of asiatic acid were investigated by adding 50 μM of glycyrrhizin or verapamil to rat liver microsomes and preincubating them for 30 min at 37 °C, followed by the addition of asiatic acid (1 μM). Aliquots of 30 μL were collected from reaction volumes at 0, 1, 3, 5, 15, 30 and 60 min; 60 μL ice-cold acetonitrile containing IS was added to terminate the reaction, and then the concentration of asiatic acid was determined using LC–MS.

The *in vitro* half-life (*t*_1/2_) was obtained using the equation: $t_{1/2}=0.693/k$; V (μL/mg) = volume of  incubation (μL)/protein in the incubation (mg); intrinsic clearance (Clint) (μL/min/mg protein)=*v* × 0.693/*t*_1/2_.

### Data analysis

The pharmacokinetic parameters, including the area under the plasma concentration–time curve (AUC), maximal plasma concentration (*C*_max_), the time for the maximal plasma concentration (*T*_max_) and the mean residence time (MRT) were calculated using the DAS 3.0 pharmacokinetic software (Chinese Pharmacological Association, Anhui, China).

The differences between the mean values were analysed for significance using a one-way analysis of variance (ANOVA). Values of *p <* 0.05 were considered to be statistically significant.

## Results

### Effects of glycyrrhizin on the pharmacokinetics of asiatic acid

**Figure 1.**
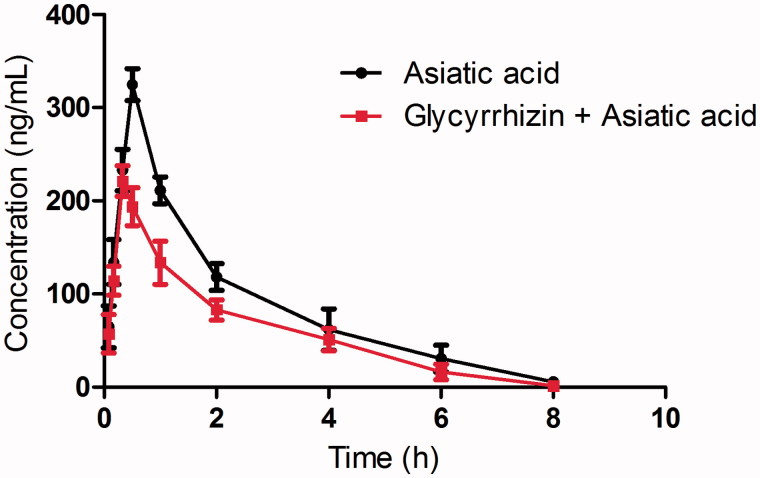
Pharmacokinetic profiles of asiatic acid in male Sprague-Dawley rats after oral administration of 20 mg/kg asiatic acid with or without glycyrrhizin (100 mg/kg/day for seven days) pretreatment. Each symbol with a bar represents the mean ± S.D. of six rats.

The mean plasma concentration–time curves of asiatic acid after oral administration of asiatic acid or oral administration of asiatic acid with the pretreatment of glycyrrhizin are presented in Figure 1. The pharmacokinetic parameters of asiatic acid were calculated using the noncompartmental method with the DAS 3.0 pharmacokinetic software (Chinese Pharmacological Association, Anhui, China). The pharmacokinetic parameters are shown in Table 1.

**Table 1.** Pharmacokinetic parameters of asiatic acid in rats after oral administration of asiatic acid (20 mg/kg; *n* = 6, mean ± S.D.) with or without treatment of glycyrrhizin.

Parameter	Control	Pretreatment of glycyrrhizin
*T*_max_ (h)	0.54 ± 0.04	0.36 ± 0.02
*C*_max_ (ng/mL)	324.67 ± 28.64	221.33 ± 21.06*
*t*_1/2_ (h)	2.04 ± 0.32	1.21 ± 0.27
AUC_(0–inf)_ (μg h/L)	749.15 ± 163.95	496.12 ± 109.31*
Oral CL (L/h/kg)	27.59 ± 5.34	41.57 ± 9.19*

* *p* < 0.05 indicates significant differences from the control.

When the rats were pretreated with glycyrrhizin, the *C*_max_ and AUC_0–inf_ of asiatic acid decreased significantly (*p* < 0.05) compared with the control group. The pharmacokinetic results also showed that glycyrrhizin could decrease the *t*_1/2_ value and increase the oral clearance rate of asiatic acid (*p* < 0.05). These results indicated that glycyrrhizin could decrease the systemic exposure and accelerate the oral clearance rate of asiatic acid when they are co-administered.

### Effects of glycyrrhizin on the absorption of asiatic acid in Caco-2 cell transwell model

**Figure 2.**
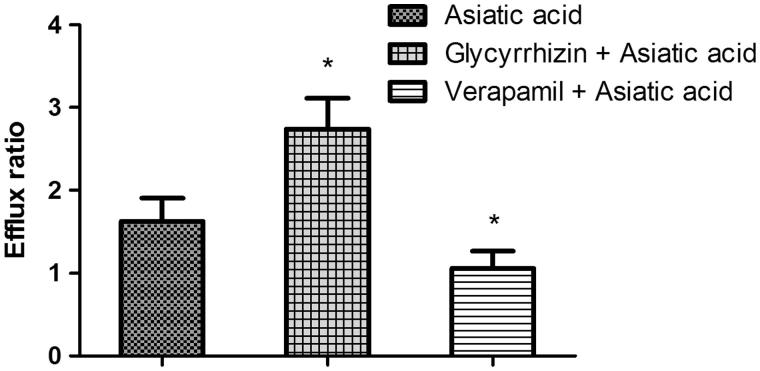
Effects of glycyrrhizin and/or verapamil on the efflux ratio of asiatic acid in the Caco-2 cell monolayer model. Each point represents the average ± S.D. of three determinations. *p < 0.05 indicates significant differences from the asiatic acid group.

To investigate the effects of glycyrrhizin on the absorption of asiatic acid, the Caco-2 cell transwell model was used. The TEER value (586.25 ± 35.81 Ω·cm^2^), paracellular flux (0.82 ± 0.12%) and the ratio of alkaline phosphatase activity (AP/BL, 6.25 ± 0.27) were all qualified for the transport studies. To validate the efflux activity of P-gp, a typical P-gp substrate digoxin was used, and the results indicated that the efflux ratio of digoxin was 10.85, which was abrogated in the presence of a typical P-gp inhibitor verapamil. After pretreatment with glycyrrhizin for 24 h, the efflux ratio of digoxin increased from 10.85 to 15.64. The results indicated that the efflux activity of P-gp was qualified for the experiment. Next, the transport of 5 μM of asiatic acid across the Caco-2 cell transwell model was investigated in this study. As shown in Figure 2, the *P_appAB_* and *P_appBA_*were 1.01 ± 0.20 × 10^−6^ cm/s and 1.65 ± 0.37 × 10^−6^ cm/s, respectively. The *P_appBA_*was much higher than the *P_appAB_*, and the efflux ratio was 1.63, which indicated that efflux transporters might be involved in the transport of asiatic acid. After that step, the transport studies were performed in the presence of glycyrrhizin or verapamil to determine its effects on the transport of asiatic acid. In the presence of 50 μM of glycyrrhizin, the *P_appAB_* decreased (0.82 ± 0.11 × 10^−6^ cm/s), whereas *P_appBA_* increased (2.25 ± 0.41 × 10^−6^ cm/s). The efflux ratio increased from 1.63 to 2.74 (*p* < 0.05). However, in the presence of verapamil (50 μM), a typical P-gp inhibitor, the efflux ratio decreased from 1.63 to 1.06 (*p* < 0.05). These results indicated that P-gp was involved in the transport of asiatic acid in the Caco-2 cell transwell model, and glycyrrhizin could enhance the efflux of asiatic acid by inducing the activity of P-gp.

### Effects of glycyrrhizin on the metabolic stability of asiatic acid in rat liver microsome incubation systems

The effects of glycyrrhizin on the metabolic stability of asiatic acid were investigated using rat liver microsome incubation systems. The metabolic half-life of asiatic acid was 10.02 ± 2.21 min, and the intrinsic clearance rate of asiatic acid was 138.32 ± 11.20 μL/min/mg protein, while in the presence of glycyrrhizin, the metabolic half-life (6.25 ± 1.53 min) was decreased, and the intrinsic clearance rate (221.76 ± 16.85 μL/min/mg protein) was increased. However, an CYP3A4 inhibitor, verapamil, could increase the metabolic half-life (26.51 ± 5.36 min) (*p* < 0.05) and decrease the intrinsic clearance rate (52.28 ± 7.95 μL/min/mg protein) (*p* < 0.05) of asiatic acid. The pharmacokinetic experiments revealed that glycyrrhizin could decrease the *t*_1/2_ value and increase the oral clearance rate of asiatic acid, and these results were verified by using the rat liver microsome incubation experiments, as glycyrrhizin could accelerate the metabolism of asiatic acid and increase the intrinsic clearance rate in rat liver.

## Discussion

The pharmacokinetic experiments showed that glycyrrhizin could decrease the systemic exposure and increase the oral clearance rate of asiatic acid when they are co-administered in rats. Zhao et al. (2017) have reported that when co-administered with glycyrrhizin in rats, the systemic exposure of pristimerin will be decreased. Yan et al. (2017) have also indicated that glycyrrhizin could decrease the systemic exposure of celastrol when they are co-administered. Previous studies have also reported that the mechanism of licorice to reconcile various drugs may be related to induction of CYP3A4 or P-gp expression, thereby accelerating the metabolism or efflux of co-administered TCM or toxic constituents in other TCMs and decreasing the toxicity thereafter (Chen et al. 2009; Tu et al. 2010; Wang et al. 2013; Xu et al. 2013; Zhang et al. 2014). Since asiatic acid is substrate of P-gp and CYP450 enzymes (Hien et al. 2010; Liang et al. 2012), we speculated that glycyrrhizin might decrease the systemic exposure of asiatic acid by enhancing P-gp-mediated drug efflux or CYP450-mediated metabolism.

To investigate its potential mechanism, the Caco-2 cell transwell model was used. The results indicated that P-gp was involved in the transport of asiatic acid, and glycyrrhizin could increase the efflux of asiatic acid by inducing the activity of P-gp. The pharmacokinetic experiments also revealed that glycyrrhizin could decrease the *t_1/2_* value and increase the oral clearance rate of asiatic acid, and these results were verified by using the rat liver microsome incubation experiments, as glycyrrhizin could increase its intrinsic clearance rate. These results indicate that the drug–drug interaction between glycyrrhizin and asiatic acid might appear when they were co-administered. Feng et al. (2015) have also indicated that drug interactions should be cautioned when medications co-administered with glycyrrhizin or related products.

In conclusion, this study suggests that co-administration of glycyrrhizin and asiatic acid could decrease systemic exposure of asiatic acid. The *in vitro* experiments showed that glycyrrhizin might exert these effects by decreasing the absorption of asiatic acid by inducing the activity of P-gp in the intestine or by accelerating its metabolism by inducing the activity of CYP450 enzymes. These results remind us that the dose of asiatic acid should be adjusted when asiatic acid and glycyrrhizin are co-administered in the clinic.
